# Diversity of 16S-23S rDNA Internal Transcribed Spacer (ITS) Reveals Phylogenetic Relationships in *Burkholderia pseudomallei* and Its Near-Neighbors

**DOI:** 10.1371/journal.pone.0029323

**Published:** 2011-12-14

**Authors:** Andrew P. Liguori, Stephanie D. Warrington, Jennifer L. Ginther, Talima Pearson, Jolene Bowers, Mindy B. Glass, Mark Mayo, Vanaporn Wuthiekanun, David Engelthaler, Sharon J. Peacock, Bart J. Currie, David M. Wagner, Paul Keim, Apichai Tuanyok

**Affiliations:** 1 Department of Biological Sciences, Center for Microbial Genetics and Genomics, Northern Arizona University, Flagstaff, Arizona, United States of America; 2 Division of Pathogen Genomics, The Translational Genomics Research Institute North, Flagstaff, Arizona, United States of America; 3 Bacterial Zoonoses Branch, Division of Foodborne, Bacterial and Mycotic Diseases, National Center for Zoonotic, Vector-Borne and Enteric Diseases, Centers for Disease Control and Prevention (CDC), Atlanta, Georgia, United States of America; 4 Charles Darwin University, Menzies School of Health Research, Darwin, Northern Territory, Australia; 5 Faculty of Tropical Medicine, Mahidol University, Bangkok, Thailand; Columbia University, United States of America

## Abstract

Length polymorphisms within the 16S-23S ribosomal DNA internal transcribed spacer (ITS) have been described as stable genetic markers for studying bacterial phylogenetics. In this study, we used these genetic markers to investigate phylogenetic relationships in *Burkholderia pseudomallei* and its near-relative species. *B. pseudomallei* is known as one of the most genetically recombined bacterial species. *In silico* analysis of multiple *B. pseudomallei* genomes revealed approximately four homologous rRNA operons and ITS length polymorphisms therein. We characterized ITS distribution using PCR and analyzed via a high-throughput capillary electrophoresis in 1,191 *B. pseudomallei* strains. Three major ITS types were identified, two of which were commonly found in most *B. pseudomallei* strains from the endemic areas, whereas the third one was significantly correlated with worldwide sporadic strains. Interestingly, mixtures of the two common ITS types were observed within the same strains, and at a greater incidence in Thailand than Australia suggesting that genetic recombination causes the ITS variation within species, with greater recombination frequency in Thailand. In addition, the *B. mallei* ITS type was common to *B. pseudomallei*, providing further support that *B. mallei* is a clone of *B. pseudomallei*. Other *B. pseudomallei* near-neighbors possessed unique and monomorphic ITS types. Our data shed light on evolutionary patterns of *B. pseudomallei* and its near relative species.

## Introduction

Utilizing length polymorphisms within the 16S-23S ribosomal DNA internal transcribed spacer (ITS) as genetic markers, aka PCR ribotyping, was developed by Kostman and colleagues [1]. This technique was first used as an alternative method to traditional 16S ribosomal DNA (rDNA) ribotyping for detecting epidemiological differences between *Burkholderia cepacia* strains [1]. The technique involves PCR of the 16S-23S internal transcribed spacer (ITS), aka the 16S-23S intergenic spacer region, and assigns phylogenetic groupings based on length polymorphisms, sequence differences or restriction patterns in the ITS [2], [3], [4]. Detailed sequence analyses of the ITS in many bacteria reveal its functional role in ribosomal RNA (rRNA) maturation and transcriptional anti-termination [5], [6], [7]. In many bacterial species the ITS contains coding sequences for tRNA genes. Consequently, the region is under selective pressure and is fairly conserved, even across bacterial species. Multiple rRNA operons (*rrn*) have been confirmed within single bacterium, sometimes as many as 15, which often display intragenomic heterogeneity in ITS type [8], [9], [10]. Copy number of the *rrn* operon has been positively associated with bacterial resource utilization and growth rate [11]. ITS variation has been attributed to intragenic evolution as well as the insertion or deletion of sequence blocks; because of this, the ITS has been referred to as a mosaic sequence [9], [12]. High intra- and interspecies homology of conserved blocks has led to the conclusion that the primary source of ITS variation is homologous recombination [9], [13].

ITS typing has since been applied successfully to a number of bacterial systematic inquiries, e.g., subtyping of *Clostridium difficile* [12], *Prochlorococcus* and *Synechococcus* [7], and *Acinetobacter calcoaceticus*-*A. baumannii* complex [14]. Widely applied elsewhere, this technique has only been minimally investigated in *Burkholderia pseudomallei* [15], [16], and only for the purposes of clinical diagnostics.

*B. pseudomallei* is the causative agent of melioidosis. Melioidosis is primarily acquired environmentally via inhalation, percutaneous inoculation or ingestion, and is associated with a variety of clinical outcomes, ranging from latent or subclinical infection to pneumonia, which often progresses to systemic sepsis and death [17]. The disease is endemic in tropical regions of northern Australia and northeastern Thailand and is associated with mortality rates of 20% and 50%, respectively [18], [19]. Advances in *B. pseudomallei* diagnostics indicate greater geographic distribution than previously thought, and the bacterium has been categorized as sporadic in other tropical regions, such as in Africa and South America [20]. Additionally, the bacterium is categorized by the Centers for Disease Control and Prevention (CDC) as a Category B Select Agent, because of its amenability to deliberate large-scale dissemination and high morbidity and mortality [21].

A high degree of genomic variability exists between strains, complicating genetic and phylogenetic studies. The *B. pseudomallei* genome is composed of ∼7.2 Mbp on two chromosomes and exhibits a relatively high GC-content of 68% [22]. The four completed *B. pseudomallei* whole genome sequences (strains K96243, 1710b, 1106a, and MSHR668) had four *rrn* operons. This genome is described as an “open genome,” with variability attributed mostly to genomic islands and islets, paralogous genes and indels [23], [24]. Such variation is mediated by the interaction of insertion sequence elements and DNA mobilizing genes such as integrases and recombinases, or by horizontal gene transfer and subsequent homologous recombination [24], [25], [26]. *B. pseudomallei* has been identified as one of the most genetically recombined bacterial species [26]. Because of this high level of variation, finding reliable molecular targets to identify strains and their relationships remains difficult. High rates of recombination have complicated multi-locus sequence typing (MLST) and multi-locus variable number tandem repeat analysis (MLVA) schemes in the species [27], [28] and may also be responsible for ribotype variability observed in restriction fragment length polymorphism (RFLP) [29] and pulsed-field gel electrophoresis (PFGE) [30] studies. In contrast, sequence analysis of the 16S rRNA gene showed nearly 100% conservation, often differing by only one single nucleotide polymorphism, making it difficult to resolve isolates using this locus [31].

The objective of this study was to elucidate phylogenetic relationship within *B. pseudomallei* and among closely related species using a high-throughput ITS typing scheme across a large, geographically and epidemiologically diverse strain panel. This adds to the scientific understanding of bacterial ITS diversity, as well as the ever-increasing knowledge of *B. pseudomallei* diversity, and may be used in conjunction with other typing studies to inform accurate phylogenetic conclusions within the species. Here, we also report that ITS typing can be complementary to MLST, and used in phylogenetic analysis of *B. pseudomallei* and its near neighbors.

## Methods

### Bioinformatics analysis

**Figure 1:**
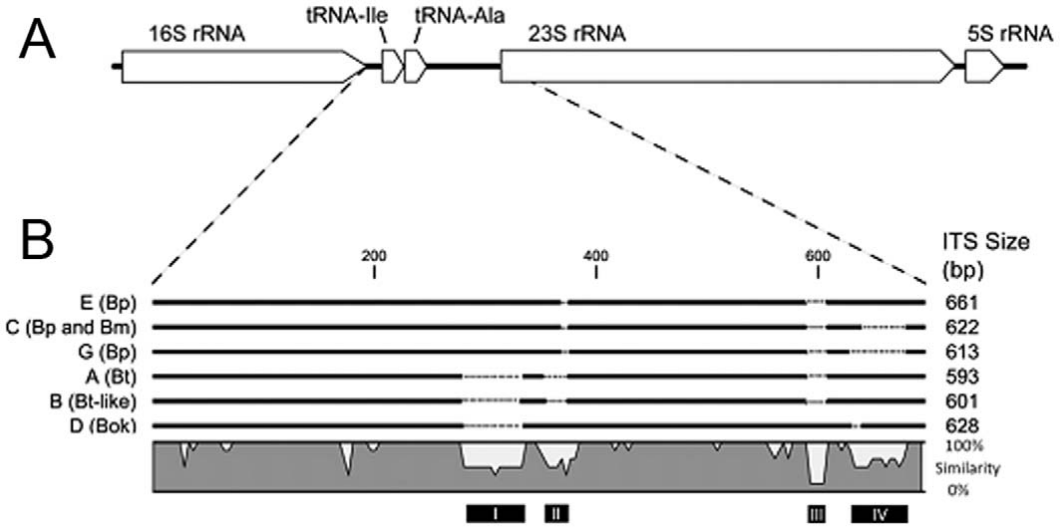
Genomic layout of the *Burkholderia* ITS. Panel A shows diagram of bacterial rRNA operon in *Burkholderia* species, demonstrating the specific location of the 16S-23S ITS. Panel B shows low resolution genomic alignment generated from consensus sequences of known ITS sequences, to highlight 1) sequence similarity among ITS types and 2) regions of difference between ITS types. Sequence labels include ITS code followed by species designation as follows: Bp (*B. pseudomallei*), Bm (*B. mallei*), Bt (*B. thailandensis*), Bt-like (*B. thailandensis*-like, referring to the reference strain MSMB43) and Bok (*B. oklahomensis*). ITS sizes are described at the right. Black bars at the bottom highlight major regions of variability I, II, III and IV that contribute to ITS fragment length polymorphisms, which are further demonstrated in the sequence conservation plot. Note that peaks outside of regions I, II, III and IV correspond to rare frame-shift and single nucleotide mutations, and are primarily the result of alignment with *B. oklahomensis*, whose ITS sequence is most divergent. Dotted lines represent missing sequences.

NCBI's BLASTN algorithm was used in order to discover variability in ITS size and in *rrn* operon copy number in many *Burkholderia* species' whole genome sequences (Fig. 1; Table S1). Variability in *rrn* genomic location was assessed in ten *B. pseudomallei* genomes, in both completed genomes and whole genome shotgun sequences (Table 1) that had been assembled using the Artemis Comparison Tool [32]. To discover the causative elements of length polymorphisms between ITS types, ITS sequences were aligned using the ClustalW multiple alignment method in BioEdit [33]. To deduce ITS sequence identity among types, a sequence distance matrix was generated using MegAlign software in the Lasergene suite version 8.0.2 (DNASTAR, Inc), from which average pair-wise sequence similarity values (*d*-values) were produced. To deduce sequence identity between types, non-variable regions from each ITS type were extracted, concatenated, and average pair-wise similarities calculated as above. For generation of the ITS alignment, consensus ITS sequences for each type were generated using BioEdit, and subsequently aligned using the slow/accurate multiple alignment tool in the CLC Sequence Viewer version 6.0 (CLC bio A/S).

**Table 1:** The genomic locations of *rrn* operons are conserved in *B. pseudomallei*.

		Chromosome 1			Chromosome 2		
Strain Name	No. *rrn* loci	Locus A	Locus B	Locus C	Locus D	Genome Status	GenBank Accession No.(s)
K96243	4	Ref.	Ref.	Ref.	Ref.	Completed	BX571965, BX571966
1710b	4	+	+	+	+	Completed	CP000124, CP000125
1106a	4	+	+	+	+	Completed	CP000572, CP000573
MSHR0668	4	+	+	+	+	Completed	CP000570, CP000571
MSHR0305	4	+	+	+	+	Shotgun†	AAYX00000000
MSHR0346	4	+	+	+	+	Shotgun†	NC_012695, NC_ACOJ00000000
406e	4	+^t^	+	+	+	Shotgun†	AAMM00000000
Pasteur 52237	3	+^t^	+	-	+	Shotgun†	AAHV00000000
S13	3	+	+	-	+	Shotgun†	AAHW00000000
MSHR1655	3	+	+	-	+	Shotgun†	AAHR00000000

**Footnote:** Here, *rrn* genomic distribution was characterized in *B. pseudomallei* by comparing both complete and incomplete^†^ whole genome sequences to the completed strain, K96243. Prior to comparison, contigs from incomplete sequences were assembled using the Artemis Comparison Tool to generate artificial chromosomes [32]. The presence of only three *rrn* operons may be a result of incomplete genomic coverage or imperfect assembly. In some cases^t^, *rrn* operons were present in translocated and/or inverted genomic regions, in which case the genomic location is still considered to be identical to the reference sequence.

### DNA samples and bacterial strains

DNA samples were obtained from different sources (see Table S2) and were prepared by several different methods. Essentially any genomic DNA preparation sufficient in yield and quality was interrogated. All putative *B. pseudomallei* isolates were cultured on selective Ashdown agar prior to DNA extraction, and were further confirmed as *B. pseudomallei* using species-specific SYBR-Green (Applied Biosystems, Inc.) real-time PCR assays, including the TTS1 [34] and the multiplex assay targeting either the *B. thailandensis*-like flagellar gene cluster (BTFC) or *Yersinia*-like fimbrial (YLF) gene cluster [35]. The identity of *B. thailandensis* isolates used were confirmed by targeting the *cheB* gene in the *B. thailandensis* homolog of BTFC [35], and *B. mallei* isolates were confirmed by targeting the *bimA* gene [36]. For the *B. thailandensis-*like MSMB43 strains and *B. oklahomensis* strains used, characterization and species confirmation was performed at the United States CDC laboratory and included biochemical testing, 16S rDNA sequencing, MLST typing and DNA/DNA hybridization [37], [38].

### PCR analysis

To interrogate ITS fragments sizes in many *Burkholderia* species, PCR primers were designed to conserved regions of the 16S and 23S rRNA genes which flank the ITS. The ITS was amplified using approximately 500 picograms of template DNA in 10 µL reactions of the HotStarTaq Master Mix (QIAGEN) and the PCR primers GTW_For (5′- GTGAAGTCGTAACAAGGTAGCCGT-3′) and, to facilitate capillary electrophoresis, a 6-carboxyfluorescein labeled reverse primer, GTW_Rev_FAM (5′-/56-FAM/ AAGGCATCCACCACATGCACTT-3′) at a final concentration of 0.2 µM each. Thermal cycling was performed using an MJ Research PTC-0200 DNA Engine (BioRad) by the following parameters: 95°C for 15 min, followed by 35 cycles of 95°C for 30 sec, 62°C for 30 sec, 72°C for 1 min and a final extension of 72°C for 10 min.

To prepare PCR products for capillary electrophoresis, amplicons were diluted 1∶150 in Type I water and 1 µL each was denatured in reactions of 13.875 µL Hi-Di Formamide (Applied Biosystems, Inc.) and 0.125 µL GeneScan – 1200 LIZ Size Standard (Applied Biosystems, Inc.) at 95°C for 4 min. Products were then electrophoresed on an Applied Biosystems 3730xl DNA Analyzer, and ITS fragment sizes were auto-analyzed and binned using GeneMapper Software Version 4.0 (Applied Biosystems, Inc.).

To facilitate rapid screening of *B. pseudomallei* ITS types, TaqMan real-time PCR assays were developed and validated against a subset of those isolates screened (Text S1).

### Statistical analysis

We used chi-square tests of association to examine relationships of ITS incidence in two endemic populations, Australia and Thailand. Three degrees of freedom were used in the analysis.

### ITS sequencing

Twenty-two representative isolates (Table S1) were selected for ITS sequencing in order to confirm 1) sequence identity among identical ITS fragment sizes and 2) sequence conservation regardless of the geographic origin of the allele or its presence in a mixed array. For sequencing, PCR of the ITS was performed as before with the exception that an unlabelled reverse primer GTW_Rev (5′-AAGGCATCCACCACATGCACTT-3′) was used. PCR products were purified using ExoSAP-IT according to the manufacturer's protocol, with the difference that half of the recommended solution was used (USB Corporation; Affymetrix, Inc.). Cycle sequencing was carried out using the Big Dye Terminator (BDT) version 3.1 kit (Applied Biosystems, Inc.), according to the manufacturer's protocol. For isolates that possessed a single ITS type, direct cycle sequencing was performed with purified amplicons as template DNA.

### GenBank ITS sequence submission

Using the Sequin Application (http://www.ncbi.nlm.nih.gov/Sequin/), all ITS sequences were annotated and submitted to GenBank under the accession numbers FJ981703 – FJ981726 (Table S1).

## Results

### Bioinformatics analysis

In order to assess *rrn* genomic distribution, we compared genomes from nine *B. pseudomallei* strains to the reference strain K96243, and found *rrn* genomic locations to be conserved (Table 1). We found four homologous *rrn* operons in all completed *Burkholderia* genomes analyzed although in some unfinished *B. pseudomallei* genomes, only three loci were observed. This may be due to incomplete genomic coverage or imperfect assembly. The only differences in location were explained by rare large-scale genomic rearrangement events (Table 1).

*In silico* analysis of various *Burkholderia* genomes revealed variation in the size of ITS regions within and among species. To facilitate comparisons, ITS fragment sizes were assigned alpha codes (Table 2). When searched, four completed *B. pseudomallei* genomes were found to possess one of two ITS types (type C or E) or had a mixture of the two ITS types distributed on the four *rrn* operons (type CE).

**Table 2:** *Burkholderia* ITS coding scheme and average pair-wise similarities.

ITS Type Code	ITS Size (bp)	Representative Organism(s)	Similarity (n = No. of Sequences)	No. Strains Typed
A	593	*B. thailandensis*	0.998 (n = 8)	88
B	601	Australian *B. thailandensis*-like (MSMB43)	0.998 (n = 2)	2
C	622	*B. pseudomallei and B. mallei*	0.999 (n = 18)	408
D	628	*B. oklahomensis*	n/a (n = 1)	2
E	661	*B. pseudomallei*	0.995 (n = 20)	523
F	553	*B. cepacia* *	n/a - not sequenced	2
**G	613	*B. pseudomallei*	0.994 (n = 7)	15
CE	622, 661	*B. pseudomallei*	n/a - mixed ITS	289
**GE	613, 661	*B. pseudomallei*	n/a - mixed ITS	2
**GC	613, 622	*B. pseudomallei*	n/a - mixed ITS	1

**Footnote:** *These are uncharacterized members of the *Burkholderia cepacia* complex.

**These ITS types were discovered in this work.

ITS types were interspecific in *B. pseudomallei* near-neighbors. All four *B. mallei* genomes possessed ITS type C that was virtually identical to the *B. pseudomallei* homolog. Genomes of *B. thailandensis* E264, *B. thailandensis-*like strain MSMB43, and *B. oklahomensis* strains C6786 and E0147 possessed an ITS fragment size distinct from any other *Burkholderia* species analyzed, designated as ITS types A, B and D, respectively (Table 2).

### PCR results

PCR screening of *Burkholderia* ITS types confirmed trends observed through *in silico* analysis of whole genome sequences and facilitated the discovery of a third major *B. pseudomallei* ITS type (ITS type G). Comprehensive typing data is presented in Table S2. All tested *B. pseudomallei* isolates possessed either a single homogeneous ITS type (either C, E, or G) or a heterogeneous mixture of two ITS types arrayed in unknown orientation in their four *rrn* operons (Table 2). The predominant ITS types found in 1,191 tested *B. pseudomallei* strains were C and E, accounting for 30.31% and 43.91% of ITS types, respectively. ITS type G represented a third major type of *B. pseudomallei* and was rare, accounting for 1.26% of isolates tested. Pair-wise mixtures were observed of all single ITS types, including CE (24.27%), GC (0.08%) and GE (0.17%).

Additionally, ITS typing confirmed species classifications of *B. pseudomallei* phylogenetic near-neighbors (Table S2). ITS types were not species specific based upon testing *B. thailandensis* (n = 88), *B. mallei* (n = 47), *B. oklahomensis* (n = 4) and Australian *B. thailandensis*-like (n = 2) strains. All *B. mallei* strains possessed only ITS type C, a type common to *B. pseudomallei*. Two strains of *B. cepacia* (Table S2) were found to possess ITS type F, but these are likely clonal members of the *Burkholderia cepacia* complex [39]. Other members of the complex were screened and a high degree of diversity was found (data not shown). Therefore, allele F should not be considered as a marker for the diverse “Pseudomallei” phylogenetic group.

### Worldwide distribution of *B. pseudomallei* ITS types and other correlates

**Figure 2:**
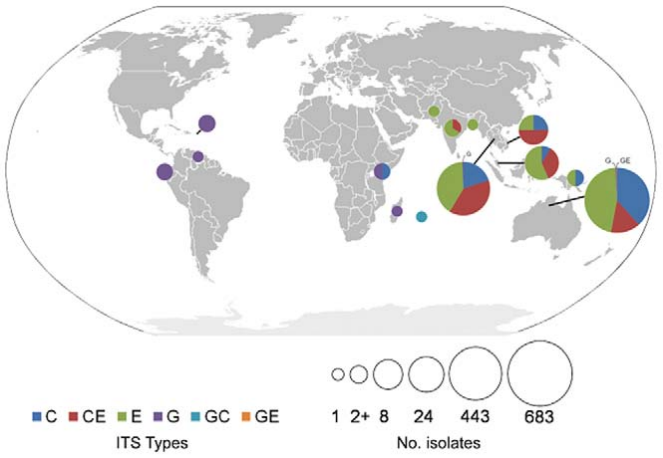
Geographic distribution of *B. pseudomallei* ITS types. ITS types are color coded. Pie chart sizes reflect the number of isolates characterized. Samples taken from sporadic melioidosis regions, as in Africa, Central and South America, frequently possessed ITS type G, shown in purple. Endemic melioidosis areas show a high degree of ITS diversity, but possessed very few isolates with the G allele (0.88% of Australian isolates and 0.68% of Thai isolates).

**Figure 3:**
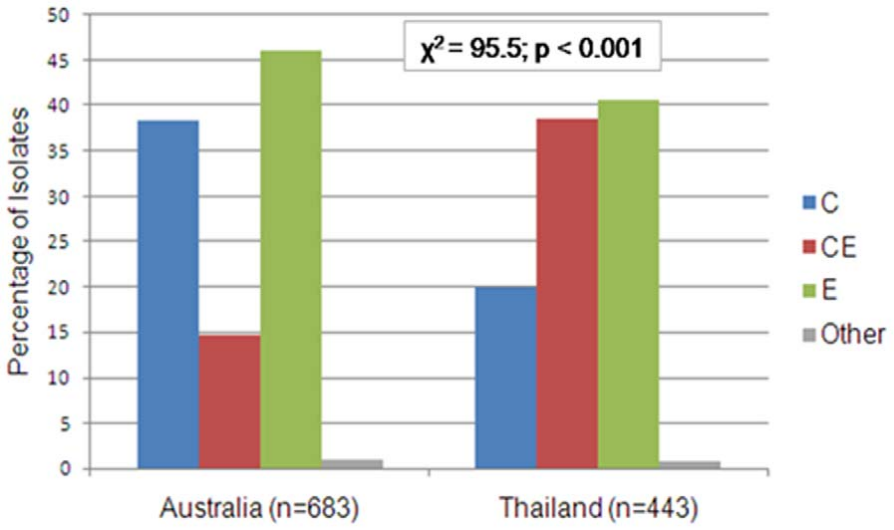
Frequency distribution plot of *B. pseudomallei* ITS types in the primary regions of endemicity, Australia and Thailand. ITS types C, E and CE were observed most frequently in these regions, while other ITS types were rarely observed. ITS type CE was observed 24% more often in Thailand as compared to Australia.

*B. pseudomallei* ITS types correlated with their geographic origins (Fig. 2). In endemic populations such as northern Australia and Southeast Asia, ITS types C, E and hybrid CE dominated, whereas the less common type G was associated with isolates found in sporadic melioidosis regions such as Africa and South America. In fact, eight out of nine samples isolated from non-endemic locations possessed a G allele. The G allele was rarely observed in endemic populations, occurring 0.88% and 0.68% in Australian and Thai isolates tested, respectively. Interestingly, isolates possessing type CE were much more common in Thailand compared to Australia, whereas ITS type E was greatest in the Australian population (Fig. 3).

Other ITS correlations were investigated. For example, many isolates that were ITS typed had also been MLST typed, and single sequence types (STs) were rarely composed of single ITS types (Table S2). In some cases, however, MLST sequence types had conserved ITS types, as with the dominant Australian sequence types ST109 and ST132, which exclusively possessed ITS types E and C, respectively. Such a result was rarely seen in Thailand.

Epidemiological correlations with ITS type were investigated, but no consistent pattern was observed when stratifying isolates by their sources (clinical versus environmental). This may be due to the recombining nature of this locus and a lack of cause-and-effect relationships between virulence and these *rrn* alleles (data not shown). It is likely that ITS alleles are frequently mobilized in the environment, therefore confounding epidemiological and phylogenetic investigations using this particular marker.

### ITS sequence analysis

DNA sequencing of representatives from each ITS type (Table S1) confirmed the near identical DNA sequence similarity among ITS types regardless of the strain's geographic origin or mixed ITS status. Pair-wise sequence similarities (*d*) among each ITS type ranged from *d* = 0.994 (ITS type G) to *d* = 0.999 (ITS type C) (Table 2). Alignment of consensus sequences from each ITS type by the multiple alignment method revealed high sequence similarity between ITS types (*d* = 0.97) with the exception of four variable regions (Var. I –IV), which are the sole contributors to ITS fragment length polymorphism (Fig. 1B).

*Burkholderia* species differed in specific ways within these variable regions (Fig. 1B). Variable regions I, II and III represent variation between different *Burkholderia* species, whereas variable region IV represents variation within *B. pseudomallei*. For example, all *B. pseudomallei* possessed conserved sequences for Var. I – II, lacked Var. III, and Var. IV variation was due to presence, absence, or length variation. However, the *B. thailandensis* ITS type A lacked sequences for all variable regions except Var. IV. ITS type B in Australian *B. thailandensis*-like MSMB43 appeared to be very similar to *B. thailandensis*, except that the lengths of missing sequence in Var. I and II were less than in ITS type A. The *B. oklahomensis* ITS type D was most divergent, lacking Var. I sequence, but possessing Var. II and III sequences, with an additional small deletion in Var. IV. Note that Var. IV sequence in *B. oklahomensis* was divergent from Var. IV sequence in *B. thailandensis*, *B. thailandensis*-like MSMB43 or *B. pseudomallei* (data not observable in Fig. 1).

## Discussion

### Insights from *Burkholderia* ITS typing

In this study, we sought to characterize the distribution of ITS differences in *B. pseudomallei* and its near-neighbors. Our ITS data indicate the existence of three major ITS types of *B. pseudomallei*. A high rate of recombination [26], [28], [40] together with mobilizing genetic elements [24], [25] has made the *B. pseudomallei* genome extremely diverse, and thus phylogenetic assignments are complicated by homoplasy. Standard 16S rDNA typing has been useful in identifying the genus and species, but the gene is too conserved to resolve *B. pseudomallei* populations [31]. ITS polymorphisms observed within *B. pseudomallei* appear to be a result of genetic recombination. Therefore, we hypothesize that single ITS types represent ancestral states on which genomic diversity has been built in the species.

Strains with mixed ITS types are most likely the result of contemporary recombination events between strains rather than being generated within a single bacterium by current *in situ* mutation events. ITS mobilization, a lateral gene transfer, has been hypothesized in other bacteria, and has been associated with homologous recombination between identical sequence blocks [12], [13], including potentially conserved tRNA genes. In fact, tRNA genes serve as “hot spots” for site-specific recombination events in other loci [24]. The species itself is prone to recombination, as found in MLST studies in which new sequence types were more commonly the result of mixing of existing alleles rather than intragenic mutations [28], [40]. The homogenous nature of lesser-recombining *B. pseudomallei* near-neighbor ITS types also supports the recombination hypothesis (Table 2).

### ITS typing and molecular epidemiology

ITS mixed types were found significantly more often in Thailand than in Australia, as evidenced by the 24% greater incidence of ITS type CE (Fig. 3). This observed high incidence of mixed types is consistent with a study suggesting higher rates of recombination in relation to mutation in Southeast Asian strains [26]. Increased soil bacterial loads likely increase the rate of contact and therefore genomic sharing between strains, and Thai soil has been found to harbor significantly more viable *B. pseudomallei* than Australian soil [41], [42], [43]. Additionally, *B. pseudomallei* incidence has been linked with soil disturbance and agriculture [41], which are commonplace activities in the Thai rice farming industry. Such ITS mixing may help to explain the low levels of association between ITS types and MLST types, especially in the Thai population.

ITS types C, E and CE represent most endemic isolates, whereas type G encompasses less than 1% of Thai or Australian isolates but is the primary type in sporadic isolates (Fig. 2). It remains unclear whether the former are simply more fit than the latter to survive in endemic regions, or whether a sampling bias exists in our isolate collection. Further ecological and functional studies, in conjunction with more thorough sampling, will be needed to elucidate this. Less clear is why sporadic *B. pseudomallei* isolates almost always possessed ITS type G. Although our sample size of sporadic isolates was small (n = 9), sampling bias is unlikely because of the high degree of association, even from multiple locations. This dispersal pattern appears to be a genetic bottleneck of unknown cause.

Dispersal by human trade is one explanation for the disparate distribution of *B. pseudomallei* ITS type G. Because genomic evidence indicates Australia as the founding population [26], [28], [35], [44], worldwide seeding from this or another endemic area is plausible. Indeed, the G allele is present in the Australian population, albeit minimally. The genotype may have accessed sporadic melioidosis locations via multiple exports from the same location, or via a single exporter that travelled to several ports worldwide. Trading of potted tropical plants may have served as the perfect temporary habitat for *B. pseudomallei*. If true, it is possible that many ITS types were exported, and that ITS type G was most fit to survive in sporadic locations. Such isolates, then, would be less fit to survive in endemic areas, which is consistent with the low incidence of allele G in Australia and Southeast Asia. Surely, comparative genomic and functional studies between endemic and non-endemic isolates must be performed in order to answer questions about bacterial fitness within and outside endemic areas.

### Conservation of the ITS sequences

ITS sequence conservation is not random (Fig. 1B). Among individual ITS types, sequence identity neared 100% in all cases (Table 2). This finding makes ITS sequencing unnecessary in future studies, unless novel ITS types are discovered. Such high levels of conservation indicate selection and a functional role. In support of this hypothesis, other studies have characterized clear roles of the ITS in rRNA processing, maturation and transcriptional anti-termination [5], [6], [7]. Additionally, tRNA gene sequences within the ITS were conserved (Fig. 1; tRNA-Ile at bp 83..142; tRNA-Ala at bp 194..253). Because sequence between ITS types was conserved when variable regions I-IV were neglected, it is likely that these conserved regions contain the functional motifs and that variable regions are extraneous. It is also possible that variable regions do serve functional roles and influence the efficiency of transcription, affecting environmental fitness and thus explaining observed bacterial niches. Indeed, *rrn* copy number has been associated with bacterial fitness [11], [45], and so it is plausible that the ITS may also play a role. Further structural and functional studies will be necessary to confirm functional roles of the *Burkholderia* ITS.

**Figure 4:**
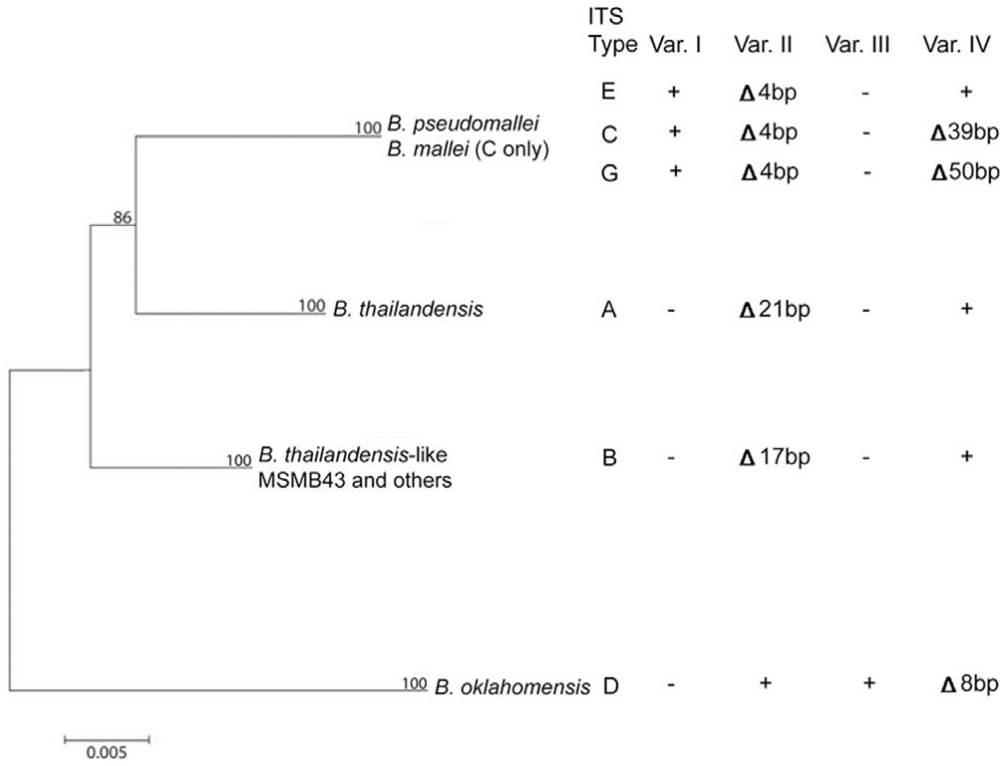
ITS variable sequence block analysis overlaid onto a *Burkholderia* MLST phylogeny (modified from Gee *et al.* [**37**]). The presence (+), absence (-) or partial absence (indicated by the Δ symbol) of variable ITS sequence blocks are described for major species-level *Burkholderia* clades, based on MLST sequence analysis. Because *B. mallei* is a clone of *B. pseudomallei* and possesses an identical ITS type, its clade was collapsed into that of *B. pseudomallei*.

In addition, ITS mutational patterns can be traced when analyzed in light of a *Burkholderia* MLST phylogeny (Fig. 4). Interspecific ITS length differences between the ITS types are attributed to specific insertion and deletion events, possibly as the result of homologous recombination between strains, which may be evolutionarily traced when compared to this phylogeny. Variable region I, for example, did not appear in *Burkholderia* species until *B. pseudomallei* speciated from a common ancestor. It is therefore likely that all *B. pseudomallei* ancestors lacked Var. I. An alternative hypothesis that a basal *Burkholderia* ancestor possessed the region, which was lost in three speciation events but kept in *B. pseudomallei,* is considerably less probable.

The most parsimonious evolutionary hypothesis for Var. II is one in which a clonal ancestor possessed sequence at that locus, which was differentially partially deleted in three speciation events: MSMB43 (Δ17 bp), *B. thailandensis* (Δ21 bp) and *B. pseudomallei* (Δ4 bp). This hypothesis is in contrast to one that suggests that the basal ancestor lacked sequence, and that identical sequence was inserted in four distinct speciation events.

### ITS and the Pseudomallei group's evolutionary patterns

ITS typing supported confirmed species classifications among species in the “Pseudomallei group” and provides further support of evolutionary hypotheses. The Pseudomallei group is one defined by NCBI's taxonomy database, and specifically includes the species studied here (http://www.ncbi.nlm.nih.gov/Taxonomy/). *B. mallei*, for example, is an obligate pathogen and has been called a clone of *B. pseudomallei,* said to have become specialized through genome reduction to occupy only the intracellular niche, rather than soil niches [46], [47]. All *B. mallei* possessed ITS type C, which was identical to the *B. pseudomallei* ortholog, supporting the species as a clone of *B. pseudomallei*, and more specifically, of the basal group of *B. pseudomallei* that possessed only ITS type C. This species showed no variation in the ITS type and had no incidence of ITS mixing (*d* = 1.00 for *B. mallei's* ITS type C), a result consistent with our previous hypothesis that inter-strain contact and subsequent horizontal gene transfer were the sources of ITS mixing. In other words, the bacterium rarely, if ever, encounters suitable gene donors with which to recombine. Because of this, *B. mallei* may be considered a clonal bacterium, while *B. pseudomallei* displays high levels of homoplastic evolution and thus cannot be considered clonal.

*B. thailandensis*, on the other hand, is more puzzling. A close genetic relative and soil commensal of *B. pseudomallei*, all *B. thailandensis* possessed ITS type A, supporting its confirmed species status [46], [48]. Like *B. mallei*, the species was completely monomorphic with regard to ITS allele, but in contrast, the bacterium certainly encounters suitable gene donors in the environment. Such aplasticity in the ITS may indicate greater overall genomic stability compared to *B. pseudomallei*, which had three possible ITS states and mixing therein. Further comparative studies in a larger *B. thailandensis* population will be necessary to confirm this hypothesis.

MSMB43, an Australian soil bacterial strain that had been described as similar to *B. thailandensis*, by 16S rDNA sequencing, MLST typing and DNA/DNA hybridization [37], and was found to possess ITS type B *in silico*. ITS sequence comparisons confirmed this relationship, as the two species possess very similar ITS types (Fig. 1B). When screened, one other isolate possessed ITS type B, MSHR1554, a strain whose MLST sequence type was found to group phylogenetically with MSMB43 [37]. The same result was confirmed in *B. oklahomensis* [38], a pathogenic soil bacterium found in the southern USA, in which all putative strains screened were found to have ITS type D. Thus, at least in non-*B. pseudomallei* isolates, ITS typing is consistent with MLST typing (Fig. 4). Because ITS type E possessed sequence at Var. IV that is most similar to the ITS types of B. thailandensis and B. thailandensis-like MSMB43 (Fig 1), we hypothesize that ITS type E represents the most ancient *B. pseudomallei* group. This inference is consistent with the result that ITS type E was most prevalent in the Australian population, which is suspected to be the founding population for all *B. pseudomallei* [44]. From ITS type E, two derivatives evolved: C and G. It is impossible to determine whether each evolved independently from ITS type E, or whether one evolved from the other as each scenario is equally parsimonious. If the latter hypothesis is correct, then type G would have evolved from type C.

In summary, this study fills an important knowledge gap in *Burkholderia* genomics and confirms trends observed in other research. By applying the widely-used technique of 16S-23S ITS typing to *Burkholderia* species we were able to demonstrate several important points. We were able to show that ancient *B. pseudomallei* was composed of three genomic groups, represented by ITS types C, E and G, and hypothesize that ITS type E is the most ancestral type. This research demonstrates that *B. pseudomallei* is highly variable in comparison to other species in the Pseudomallei group. Moreover, we demonstrate a greater level of recombination in Thai *B. pseudomallei* isolates compared to isolates from other locations, which agrees with previous findings. Regarding geographic distribution, we identified one *B. pseudomallei* group (represented by ITS type G) that correlated strongly with worldwide sporadic distribution of melioidosis, and hypothesize human travel and trade as the mechanism of this dispersal pattern. With regard to phylogenetic near-neighbors, we were able to support *B. mallei* as a clone of *B. pseudomallei*. We show that other species are evolutionarily distinct and display less genomic plasticity than *B. pseudomallei*. Increased sampling, in conjunction with comparative functional and ecological studies, will be necessary to confirm hypotheses that we present here.

## Supporting Information

Table S1 List of ITS sequences or whole-genome sources of ITS sequences used for bioinformatics analysis in this study.
(XLS)

Table S2 All ITS typing data generated in this study, including isolate contributor, geographic origin, epidemiological data and MLST data, when known.
(XLS)

Text S1 Validation of PCR-ribotyping using TaqMan real-time PCR assays.
(DOC)
