# Supplementary material for: Diversity of 16S-23S rDNA Internal Transcribed Spacer (ITS) Reveals Phylogenetic Relationships in Burkholderia pseudomallei and Its Near-Neighbors
Source: PLoS One. 2011 Dec 14;6(12):e29323. doi: 10.1371/journal.pone.0029323 (PMC3237603; doi:10.1371/journal.pone.0029323)
Supplement: Text S1 — Validation of PCR-ribotyping using TaqMan real-time PCR assays. (DOC) [file pone.0029323.s003.doc]

Text S1. Validation of PCR-ribotyping using TaqMan real-time PCR assays

To simplify ITS screening of the three major *B. pseudomallei* ITS types, we developed and validated TaqMan real-time PCR assays against a panel of diverse *B. pseudomallei* and *B. mallei* strains. Note that this screening data is not presented in this publication. Both the forward consensus primer and probe were designed to target all ITS types, and specificity is achieved through reverse primers that were designed to anneal at ITS-dependant sequences within Variable Region IV (Fig. 1). These assays have been optimized to run in singleplex, therefore three separate PCRs must be performed.

Real-time PCR was carried out in a 5.0µl reaction mixture containing 1X TaqMan Universal PCR Master Mix (Applied Biosystems, Inc.), 900nM each of a forward and reverse primer, 250nM probe, and 1.0µl DNA template at a concentration of approximately 0.5ng/µl. Thermal cycling was performed on a 7900HT sequence detection system (Applied Biosystems, Inc.) with the following conditions: 50°C for 2 min, 95°C for 10 min, and 40 cycles of 95°C for 15 sec and 60°C for 1 min.

| **Primer/probe** | **Specificity** | **Sequence** |
| --- | --- | --- |
| ITS_Consensus_For | ITS types E, C, and G | GATTGGGTTCGAGAGAACGAATC |
| ITS_E_Rev | ITS type E | GCGCTTACGAKGATCCGATAC |
| ITS_C_Rev | ITS type C | TCTCATCGACAGCCGCTACA |
| ITS_G_Rev | ITS type G | GACCCTATAACGAGTMTGTCTCGYT |
| Ribo_FAM | All ITS types | 6FAM-TTGAGTTCTCGCGTTGTG-MGBNFQ |
